# *En bloc* liver-kidney transplantation with renal artery variation using donor splenic artery and left gastric artery as inflow to the kidney: Case report

**DOI:** 10.1016/j.ijscr.2018.10.002

**Published:** 2018-10-08

**Authors:** Michelle C. Nguyen, Sylvester Black, Ken Washburn, Ashraf El-Hinnawi

**Affiliations:** Division of Transplantation, The Ohio State University Wexner Center, 395 W 12th Avenue, Columbus, OH, 43210, USA

**Keywords:** *En bloc* liver-kidney transplantation, Simultaneous liver-kidney transplantation, Renal artery variation

## Abstract

**Introduction:**

*En bloc* liver-kidney transplantation can be difficult with renal artery variations for which the risk of multiple anastomoses can outweigh the benefits.

**Presentation of case:**

This report is the first to describe an *en bloc* liver-kidney transplantation using a donor kidney with double renal arteries. The indication for a combined liver-kidney transplant was non-alcoholic steatohepatitis (NASH) cirrhosis with chronic kidney disease secondary to hypertension and diabetes compounded by hepato-renal syndrome. The explant pathology was consistent with steatohepatitis, but did have PAS/D-positive intracytoplastic globules which suggest an additional component of alpha-1-antitrypsin deficiency.

**Discussion:**

Diminished arterial inflow to the inferior renal pole was noted intraoperatively, requiring re-anastomosis of the inferior renal polar artery to the donor left gastric artery. The post-operative course was uncomplicated with patient discharge on post-operative day six.

**Conclusion:**

With increasing numbers of simultaneous liver-kidney transplants being performed, kidneys with multiple renal arteries can successfully be transplanted *en-bloc* without compromising ischemia time.

## Highlights

• En bloc liver-kidney transplant is feasible and may yield benefits without compromising allograft function and survival.
• Simultaneous venous and arterial reperfusion to en bloc liver-kidney allografts can decrease CIT and should not significantly increase WIT.
• En bloc liver-kidney transplant can offer several advantages: less operative time, decreased hernia risk, and better pain management.

## 1 Introduction

Simultaneous liver kidney transplantation (SLKT) has become a well-established option for management of patients with concurrent end stage renal and liver disease [1]. Historically, the SLKT is performed as a single contiguous procedure in which kidney transplantation (KT) immediately follows LT via a separate right (or left) lower quadrant hockey-stick incision. This often leads to longer operating and cold ischemia time (CIT). Prolonged CIT has been shown to be associated with delayed graft function (DGF), decreased graft survival, primary non-function, and early allograft dysfunction [[2], [3], [4], [5]]. In this paper, we describe a case report of a successful *en bloc* liver-kidney transplantation, a technical variant of SLKT that can potentially decrease operative time, CIT and other risks. The patient provided informed consent for publication. The work has been reported in line with the SCARE criteria [6].

## 2 Case report

The patient is a 55-year-old white male with a history of hypertension, type II diabetes, resulting in hypertensive and diabetic nephropathy, as well as NASH cirrhosis. This diagnosis was further complicated by hepatorenal syndrome, hepatic encephalopathy, esophageal varices, and ascites requiring multiple endoscopies for variceal banding and paracenteses. Abdominal imaging demonstrated cirrhosis without evidence of hepatocellular carcinoma. He did have evidence of portal hypertension, splenomegaly, and thrombosed main portal vein with poor distal reconstitution. In October of 2017, the patient received combined liver-kidney transplantation with *en bloc* graft. The donor was a brain dead 42-year-old white male. His terminal creatinine was 1.1 mg/dL and liver function tests were within normal limits.

### 2.1 Transplantation

**Fig. 1:**
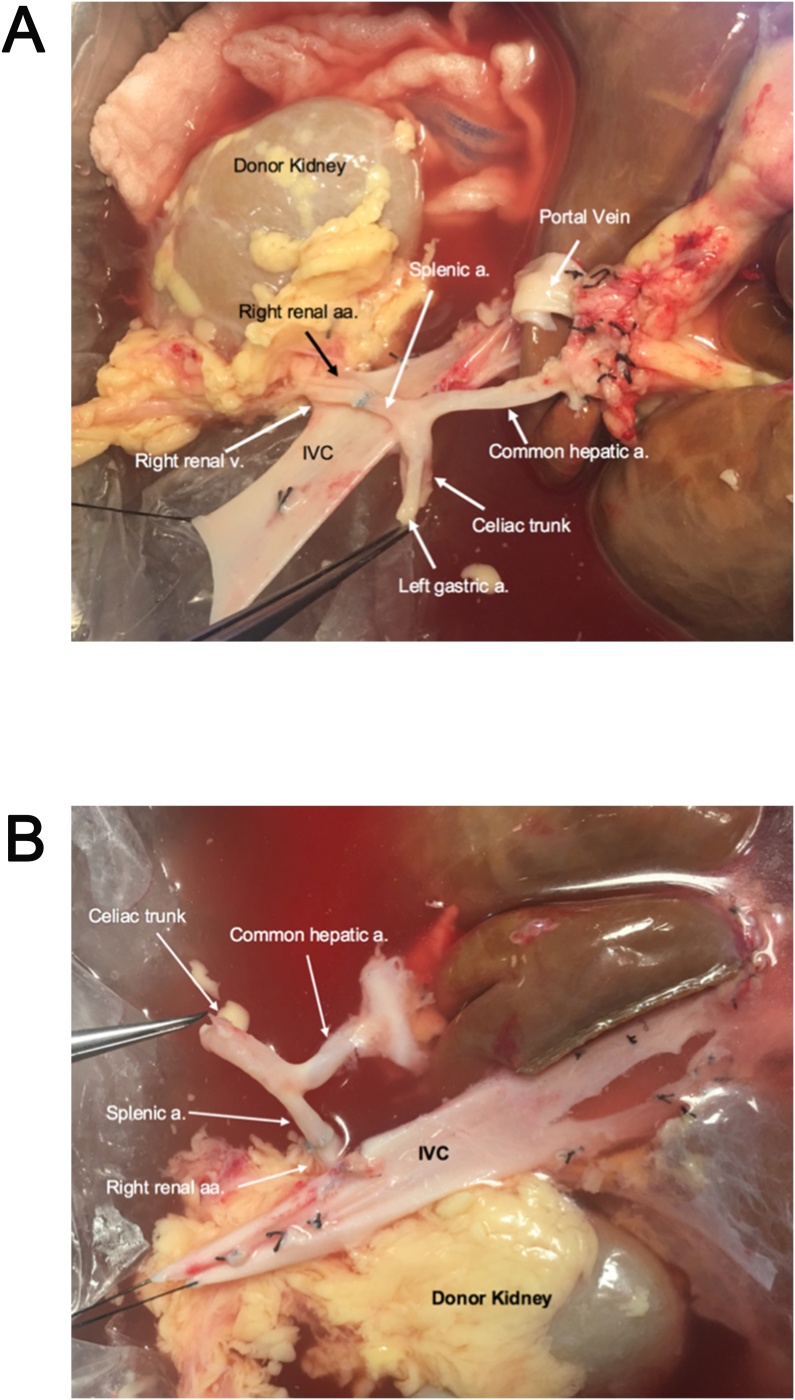
Back-table reconstruction with arterioplasty of hilar renal artery and inferior renal polar artery followed by end to end renal artery anastomosis to donor splenic artery. A: Anatomical view. B: Posterior view. a; artery, aa; arteries, v; vein.

Organ recovery was performed using standard techniques for donation after brain death (DBD) as previously described using histidine-tryptophan-ketoglutarate (HTK) solution for flushing [7]. The liver and right kidney allografts were procured *en bloc,* dividing the inferior vena cava (IVC) inferior to the right renal vein. The renal allograft did have a main hilar artery and an inferior polar artery. Standard liver and kidney back-table reconstruction was performed. However, an arterioplasty was performed on the main and lower pole renal arteries followed by end-to-end anastomosis to the donor splenic artery using 7-0 polypropylene sutures (Fig. 1 A, B).

The recipient hepatectomy was performed in the standard fashion with ligation of the hepatic arteries, common hepatic duct, and portal vein. A portal vein thrombectomy was performed using eversion technique, extended down to the confluence of the superior mesenteric vein and splenic vein resulting in adequate portal flow. The right colon was mobilized medially to identify the proximal right ureter.

**Fig. 2:**
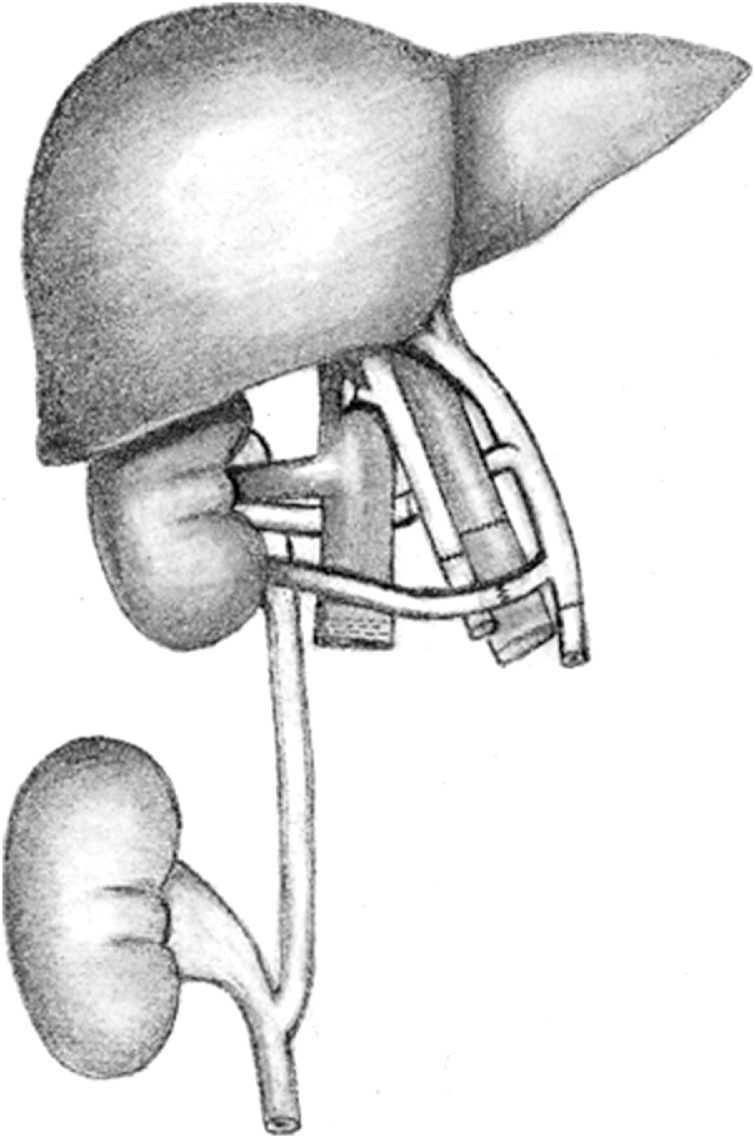
*En bloc* liver-kidney transplant: vascular, biliary and ureter anastomosis.

Transplantation was performed by modified piggy-back technique with a side-to-side cavo-caval anastomosis. The portal venous anastomosis was completed in a standard end-to-end fashion. Next, the arterial inflow to the allografts was performed by anastomosing the donor celiac artery to the recipient common hepatic artery. The renal arteries were positioned posterior to the portal vein and donor IVC. Portal venous and arterial flow was restored to the *en bloc* organs. The lower pole of the kidney did not demonstrate brisk perfusion compared to the rest of the allograft, with poor Doppler signal in the lower pole artery. Therefore, the lower pole artery was re-anastomosed to the donor left gastric artery stump anterior to the portal vein, resulting in excellent flow. Finally, an end-to-side ureteroureterostomy was performed to the recipient’s right ureter over two Double-J stents (Fig. 2). Total operative time was 8 h, wherein CIT was 4 h and warm ischemia time (WIT) was 40 min.

### 2.2 Clinical course

Postoperative liver and transplant kidney Doppler studies demonstrated normal portal vein flow (86.4 cm/s), normal hepatic artery flow with resistive index (RI) slightly elevated at 0.75, and normal arterial waveforms on kidney Doppler with RI ranging from 0.56 to 0.66 between upper pole and lower pole. Improvements in serum creatinine and liver function tests were noted immediately post-transplantation and continued to improve. The patient’s operative course was uncomplicated and he was discharged on postoperative day six. At 30-day follow up, the portal vein remained patent with normalization of hepatic artery RI at 0.68 and stable range of intrarenal arterial RI (0.49–0.53). The ureteral stents were removed by rigid cystoscopy and ureteroscopy with basket extraction by Urology 6 weeks post-transplantation.

## 3 Discussion

The results from this case report suggest *en bloc* liver-kidney transplant is technically feasible and may yield benefits to patients without comprising kidney allograft function and survival. Transplantation by this technique allows for an operation via a single incision, eliminating the risks of prolonged anesthesia. Additionally, simultaneous venous and arterial reperfusion to the *en bloc* liver and kidney allografts can decrease CIT, and when refined, should not significantly increase warm ischemia time (WIT).

The profile of kidney transplant recipients has dramatically changed over the last 30 years. More patients over the age of 65 with diabetic or hypertensive/vascular chronic kidney disease are receiving kidney transplantation [8]. Subsequently, an increasing number of recipients have associated vascular disease with infrarenal aorta and iliac calcifications which can pose significant technical challenges with classic KT techniques. In these cases, the splenic artery can serve as an alternative for arterial inflow if arterial quality is acceptable.

Gunabushanam et al. reported two successful case reports of *en bloc* liver-kidney transplants using the splenic artery as inflow to the kidney allograft. In these cases, the renal artery was positioned posterior to the portal vein and bile duct, but anterior to the donor IVC [9]. In our case, the renal artery was positioned posterior to the portal vein and donor IVC. We believe that this decreases the risk of renal arterial compression between donor IVC and portal vein, particularly given the small diameter of the lower inferior pole renal artery. Likely, positioning of renal artery in future cases will be dictated by anatomical variations.

Although *en bloc* liver-kidney transplantation may provide some advantages compared to standard SLKT, post-operative complications such as arterial inflow insufficiency, stenosis, arterial thrombosis or large postoperative hematomas can place both allografts at risk, and complications requiring reoperation can pose significant challenges in the setting of multiple fresh anastomoses in a limited working space. Therefore, careful donor and recipient selection taking into considerations of donor and recipient size match, adequate recipient hepatic artery inflow and potential need for aortic arterial conduits are important to optimize perioperative management following *en bloc* liver-kidney transplantation. Although this case report is limited by the small number of patients, we believe the case provides initial insight to guide future en bloc liver-kidney transplantations. A larger series can certainly provide more insight to the advantages of this technique.

In conclusion, when combined with careful patient selection and refined operative technique, *en bloc* liver-kidney transplantation can offer several advantages over standard procedure, including less operative and cold ischemia time, decreased hernia risk, and better pain management without compromising graft and patient survival and outcomes.

## Conflicts of interest

None.

## Sources of funding

None.

## Ethical approval

Ethical approval has been exempted by The Ohio State University.

## Consent

Written informed consent was obtained from the patient for publication of this case report and accompanying images. A copy of the written consent is available for review by the Editor-in-Chief of this journal on request.

## Author contributions

Michelle C. Nguyen: Writing – Original Draft, Writing – Review & Editing, Visualization. Sylvester Black: Conceptualization, Validation.

Ken Washburn: Conceptualization, Validation.

Ashraf El-Hinnawi: Conceptualization, Visualization, Writing – Review & Editing.

## Registration of research studies

N/A.

## Guarantor

Ashraf El-Hinnawi, MBBS, FACS.

## Provenance and peer review

Not commissioned, externally peer reviewed.
